# Adapting MultiPLe behavior Interventions that eFfectively Improve (AMPLIFI) cancer survivor health: program project protocols for remote lifestyle intervention and assessment in 3 inter-related randomized controlled trials among survivors of obesity-related cancers

**DOI:** 10.1186/s12885-022-09519-y

**Published:** 2022-04-29

**Authors:** Dori Pekmezi, Kevin Fontaine, Laura Q. Rogers, Maria Pisu, Michelle Y. Martin, Yu-Mei Schoenberger-Godwin, Robert A. Oster, Kelly Kenzik, Nataliya V. Ivankova, Wendy Demark-Wahnefried

**Affiliations:** 1 Department of Health Behavior, University of Alabama at Birmingham (UAB), Birmingham, AL USA; 2 O’Neal Comprehensive Cancer Center, UAB, Birmingham, AL USA; 3 Department of Medicine, UAB, Birmingham, AL USA; 4 Department of Preventive Medicine, The University of Tennessee Health Science Center, Memphis, TN USA; 5 Department of Health Services Administration, UAB, Birmingham, AL USA; 6 Department of Nutrition Sciences, UAB, Birmingham, AL USA

**Keywords:** Cancer, Survivorship, Older adults, Physical function, Diet, Physical activity

## Abstract

**Background:**

Scalable, multiple behavior change interventions are needed to address poor diet, inactivity, and excess adiposity among the rising number of cancer survivors. Efficacy-tested diet (RENEW) and exercise (BEAT Cancer) programs were adapted for web delivery among middle-aged and older cancer survivors for the AMPLIFI study, a National Cancer Institute-funded, multi-site, program project.

**Methods:**

Throughout the continental U.S., survivors of several obesity-related cancers are being recruited for three interconnected randomized controlled trials (RCTs). Projects 1 and 2 test 6-month diet or exercise interventions versus a wait-list control condition. Upon completion of the 6-month study period, the intervention participants receive the next behavior change sequence (i.e., diet receives exercise, exercise receives diet) and the wait-list control arm initiates a 12-month combined diet and exercise intervention. Project 3 tests the efficacy of the sequential versus simultaneous interventions. Assessments occur at baseline and semi-annually for up to 2-years and include: body mass index, health behaviors (diet quality, accelerometry-assessed physical activity/sleep), waist circumference, D3 creatine-assessed muscle mass, physical performance, potential mediators/moderators of treatment efficacy, biomarkers of inflammation and metabolic regulation, health care utilization, cost, and overall health. Four shared resources support AMPLIFI RCTs: 1) Administrative; 2) Adaptation, Dissemination and Implementation; 3) Recruitment and Retention; and 4) Assessment and Analysis.

**Discussion:**

Representing a new generation of RCTs, AMPLIFI will exclusively use remote technologies to recruit, intervene and assess the efficacy of the newly-adapted, web-based diet and exercise interventions and determine whether sequential or combined delivery works best for at-risk (older, rural, racial minority) cancer survivors.

**Trial registration:**

ClinicalTrials.gov, NCT04000880. Registered 27 June 2019.

## Background

Two-out-of-five Americans will be diagnosed with cancer during their lifetime [1]. Given improvements in early detection and treatment, most will experience “cure;” however, compared to individuals who do not have a history of cancer, cancer survivors are at increased risk for second malignancies, cardiovascular disease, and functional impairment [2–5]. Interventions are therefore needed to prevent or delay these adverse sequelae.

A physically active lifestyle and a plant-based diet, rich in whole grains, vegetables, and fruit, and low in red and processed meats, simple sugars and refined grains, are associated with avoidance of obesity and other chronic illnesses, as well as improved cancer outcomes [6]. However, many cancer survivors do not adhere to recommended diet and exercise guidelines [7]. Adherence to guidelines is even poorer in various subgroups of cancer survivors, i.e., the elderly [8], minorities [9], and those residing in rural locations [10]. Programs that provide cancer survivors with appropriate information and behavioral strategies to improve adherence to a healthy lifestyle are needed, and several efficacious interventions have been tested.

The Better Exercise Adherence after Treatment for cancer (BEAT Cancer) [11, 12] and the Reach-out to ENhancE Wellness (RENEW) [13] trials each produced durable improvements in health behaviors and outcomes among cancer survivors using in-person, as well as mail- and telephone-based approaches, respectively. With dissemination and implementation in mind, these interventions were adapted to highly scalable web-based platforms, and are now being tested in a National Cancer Institute (NCI)-funded program project called Adapting MultiPLe behavior Interventions that eFfectively Improve cancer survivor health; AMPLIFI (P01 CA229997). The aims of the AMPLIFI study include resolving a fundamental and heretofore unanswered research question [14, 15] that is key to multi-behavior diet and exercise interventions, especially those that target the large population of survivors of obesity-related cancers: What is the optimal presentation of diet and exercise content, i.e., is it best to target diet and exercise simultaneously or in sequence for at-risk (older, rural, racial minority) cancer survivors? And if in sequence, is efficacy optimized if diet is presented first then exercise second, or vice versa?

**Fig. 1:**
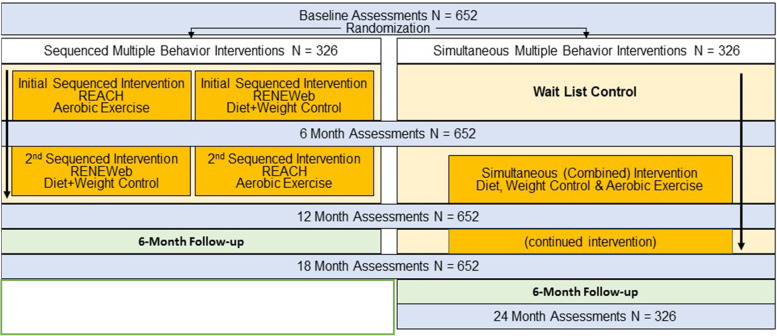
AMPLIFI study flow diagram

This report describes the methods of AMPLIFI, an ongoing study which is recruiting survivors of several obesity-related cancers (non-Hodgkin lymphoma, multiple myeloma, and cancers of the colorectum, endometrium, kidney, ovary, prostate, thyroid, and female breast) across the continental United States for three interconnected randomized controlled trials (RCT). Project 1 is an RCT of a 6-month dietary intervention to promote weight loss via caloric restriction and consumption of a nutrient-rich, low energy density diet with limited red meat, added sugars, and processed foods and ample fruits, vegetables, and whole grains. Project 2 tests a 6-month exercise intervention that encourages participation in aerobic (moderate intensity), muscle-strengthening, flexibility, and balance activities. Project 3 is an RCT in which the web-based diet and exercise intervention content and supportive materials (from projects 1 and 2) are combined and presented simultaneously versus sequentially (Fig. 1).

The main outcomes of the AMPLIFI project are diet quality, physical activity, and weight status. Secondary outcomes include body composition (muscle mass), physical performance, survivorship symptoms, health and health care utilization, and quality-of-life. Specific hypotheses for projects 1 and 2 are that intervention participants will have a significantly greater probability of reaching > 3% weight loss and national physical activity guidelines (≥150 weekly minutes of moderate-to-vigorous intensity aerobic physical activity), respectively, than participants in the wait-list control arm. Specific hypotheses for project 3 are that sequenced arm participants will have a significantly greater probability of achieving behavioral goals (improved diet quality, > 3% weight loss and ≥ 150 weekly minutes of moderate-to-vigorous intensity aerobic physical activity) than survivors randomized to the simultaneous arm, as participants (such as those who are older and who comprise a substantial proportion of the survivorship community) may find the combination of diet and exercise more overwhelming than approaching the health behavior changes one at a time [16, 17]. Significant mediators and moderators of treatment efficacy (e.g., self-efficacy, age, race, rural/urban status) will be identified to improve our understanding of how the intervention(s) work and for whom they work best. Lastly, the cost-effectiveness of interventions will be explored based on incremental costs per Quality Adjusted Life Years (QALYs). Assessments are performed exclusively via remote means, thus making AMPLIFI one of a handful of new generation RCTs for which accrual, intervention delivery and assessment are broadly scalable.

## Methods/design

### Overview

The three AMPLIFI projects include two 6-month RCTs testing diet (project 1) or exercise (project 2) interventions versus a wait-list control condition. After 6 months, diet and exercise arm participants progress to the next behavioral intervention sequence and the wait-list arm begins a 12-month combined diet and exercise intervention. Project 3 will examine data from all participants to determine the relative efficacy of the 12-month diet and exercise intervention when presented sequentially versus simultaneously to cancer survivors (Fig. 1).

Under the AMPLIFI umbrella, the three RCTs maximize economies of scale by instituting identical eligibility criteria and protocols for accrual, identical intervention design elements and outsourcing, as well as identical protocols for assessment and outcomes. Like the vast majority of behavioral intervention trials, AMPLIFI RCTs are single-blinded; assessments are performed at baseline and every 6 months by assessors who are masked to randomization status with each RCT’s primary endpoint assessed upon intervention completion and durability assessed 6 months thereafter. The three RCTs are interwoven to maximize the contribution of wait-list controls, and ability to answer the overarching research question of sequenced versus simultaneous delivery of exercise and dietary components (Fig. 1).

All three AMPLIFI RCTs are supported by a tightly-connected network of shared resources: 1. Administrative Shared Resource (disperses resources, processes incentives and manages regulatory elements); 2. Adaptation, Dissemination and Implementation (gathers formative information [18], leads qualitative and mixed methods data analyses, and facilitates the adaptation, beta-testing, standardization, process evaluation, and refinement of all intervention components), 3. Recruitment and Retention (manages contact with cancer registries, referrals, outreach with mailings and social media, screening and retention activities); and 4. Assessment and Analysis (performs baseline and follow-up assessments, implements randomization schema, and conducts data management and analyses). Regulatory elements such as clinical trial registration (NCT04000880) and single Institutional Review Board approval (UAB IRB-300002068) also are conjoined. Peer review has been completed by the funding body (National Institutes of Health). Any important protocol modifications (e.g., changes to eligibility criteria, outcomes, analyses) will be communicated to relevant parties via regular meetings with the research team, data safety and monitoring board and reports to IRB, Clinicaltrials.gov, funders, etc.

### Interventions

#### Conceptual framework

The three AMPLIFI RCTs also are bound by their common theoretical framework provided by Social Cognitive Theory (SCT), one of the more frequently used and robust health behavior theories [19]. SCT is an interpersonal model which posits that health behaviors both influence and are influenced by factors such as individual attitudes and beliefs and their environmental context. SCT has been used successfully as the underlying theory for change across many behavioral domains and diverse populations including diet [20, 21] and exercise [20–25] in rural [26, 27], older [20, 28], racial/ethnic minority groups [24, 25, 29] [26], and cancer survivors [21–23, 28], including the BEAT Cancer and RENEW trials which served as adaptation models for the AMPLIFI interventions [12, 13].

Key SCT constructs have been associated with behavior change in past studies (e.g., self-efficacy [30–33], barriers [33–36], social support [37–40]) and are targeted in the AMPLIFI assessments and interventions. Self-efficacy is promoted via self-monitoring of weight, diet quality (e.g., servings of fruits and vegetables), resistance training, and/or daily steps and incremental goal-setting with feedback. Interactive online learning sessions address common barriers to behavior change among cancer survivors (lack of time, fatigue). Facebook groups provide social support for healthy eating and active lifestyle from staff and other cancer survivors participating in the study. Moreover, the key SCT constructs directly targeted by AMPLIFI intervention strategies are assessed at multiple time points, to allow for mediation analyses.

#### Adaption process

The process of adapting the BEAT Cancer and RENEW interventions to the web-based platform used in AMPLIFI was informed by focus groups on web-based lifestyle intervention needs/preferences among cancer survivors and qualitative interviews with key stakeholders that held leadership positions in health care systems, and cancer care and support organizations [41]. The prototype interventions were created based on this input, and then beta-testing within the target population was performed. During this iterative adaptation process, the public-facing title of AMPLIFI was modified slightly to be more understandable, with the acronym more easily spelled and searched, i.e., AMPLIFY (AiM, Plan, and act on LIFestYles).

#### Content and design

The sequential (healthy diet then exercise, or vice versa) and simultaneous (diet + exercise) interventions contain identical content, based on American Cancer Society Nutrition and Physical Activity Guidelines for Cancer Survivors [6]. As such, all AMPLIFY interventions encourage a plant-based diet with ample amounts of fruits, vegetables, and whole grains, and limited sugar, refined and high-fat foods and red and processed meats. Calorie goals are set using the Mifflin-St. Jeor equation and then imposing a 500 kcal deficit for weight loss of roughly 0.5 kg/week [42]. Physical activity content is aligned with general exercise guidelines for older adults [43] and emphasizes gradually reaching the recommended levels of aerobic (150 min/week moderate-to-vigorous physical activity), muscle-strengthening (2–3 days/week) and functional (stretching and balance) exercise (3–5 days/week) for cancer survivors. To support engagement with the program, participants receive scales, portion plates, pedometers, and resistance bands, at the condition-appropriate time point/s.

As with content, the web-based platform for all AMPLIFY interventions is identical and includes a home page and sections (i.e., tabs) for My Progress, Sessions, Tools, and Support. The home page highlights intervention-appropriate “tips of the day” for staying active and eating healthy, along with quick links to complete the featured weekly educational session, track exercise and/or diet, notify AMPLIFI team of medical issues or family emergencies, check the road map for specific tasks and topics for each week, and review videos on motivational testimonials or how to handle a diet and/or exercise setback. In the My Progress section, participants track their weight, dietary consumption of specific foods (e.g., desserts and sugar sweetened beverages, servings of red or processed meats, fast food, whole grains, vegetables, fruits) and practice of specific behaviors (e.g., snacking after dinner), and/or exercise (aerobic, resistance, flexibility, and balance), as appropriate for their intervention assignment. The My Progress section also guides participants in setting goals (including tailored recommendations) and choosing a behavior change strategy from a drop-down menu of rotating content, while also providing graphical displays of behavior change progress.

The Sessions section includes intervention-appropriate interactive online learning activities related to diet and/or exercise that are released weekly (see Table 1). These sessions focus on topics such as diet and/or exercise recommendations, self-monitoring, setting Specific, Measurable, Achievable, Relevant, and Time-Bound (S.M.A.R.T.) goals, overcoming barriers, enlisting social support, dealing with relapse, cognitive restructuring, safety/injury prevention, outcome expectations, behavioral capability, role modelling, etc. The Tools section is a library of healthy lifestyle applications and resources, featuring items that range from brief tip sheets on how to select the best work-out shoes, recipe cards, and meal plans to videos of cooking demonstrations and strength training exercise tutorials. The Support section includes the link to the private AMPLIFY Facebook group, social support tip sheets, and *Ask AMPLIFY*, a library of questions and answers about making lifestyle changes. Participants are encouraged to visit the AMPLIFY website as often as possible, with regular contacts (2 email and/or text reminders per week) to alert participants to their upcoming new weekly content and progress through the 48-week interventions.

**Table 1:** AMPLIFI intervention schedule of session topics

Diet Sessions	Exercise Sessions	Diet + Exercise Sessions
Week 1: What Can I Do to Lower My Risk of Cancer?	Week 1: Physical Activity, Exercise, and Your Health	Week 1: What Can I Do to Lower My Risk of Cancer?
		Week 2: Physical Activity, Exercise, and Your Health
Week 2: Get on Track for Success!	Week 2: Understanding Your Activity Levels	Week 3: Understanding Your Activity Levels
		Week 4: Get on Track for Success!
Week 3: Be S.M.A.R.T. About Safe Weight Loss	Week 3: Achieving Goals with S.M.A.R.T. Planning	Week 5: Achieving Goals with S.M.A.R.T. Planning
		Week 6: Be S.M.A.R.T. About Safe Weight Loss
Week 4: Does Sugar Cause Cancer? The Sweet ‘n Low-down on Sugar and Fasting.	Week 4: Resistance Training for Your Health	Week 7: Resistance training for your health
		Week 8: Does Sugar Cause Cancer?
Week 5: Managing Super-Sized Temptations and Portions	Week 5: Moving Better and Making Healthy Choices Easier	Week 9: Moving Better and Making Healthy Choices Easier
		Week 10: Managing Super-Sized Temptations and Portions
Week 6: Red and Processed Meats: How Can Something So Good Be So Bad?	Week 6: Social Support for Exercise	Week 11: Social Support for Exercise
		Week 12: Red and Processed Meats: How Can Something So Good Be So Bad?
Week 7: Get the Skinny on Trimming the Fat	Week 7: Switching Up Your Routine with F.I.T.T.	Week 13: Switching Up Your Routine with F.I.T.T.
		Week 14: Get the Skinny on Trimming the Fat
Week 8: Reaping the Benefits of Whole Grains	Week 8: A Review of Your Exercise Journey	Week 15: A Review of Your Exercise Journey
		Week 16: Reaping the Benefits of Whole Grains
Week 9: Super Food Heroes: Fruits and Vegetables	Week 9: Dealing with Exercise Barriers	Week 17: Dealing with Exercise Barriers
		Week 18: Super Food Heroes: Fruits and Vegetables
Week 10: Have Concerns About Pesticides that have been Bugging You?	Week 10: Finding Time for Exercise	Week 19: Finding Time for Exercise
		Week 20: Have Concerns About Pesticides that Have Been Bugging You?
Week 11: Too Pooped to Make Healthy Diet Choices?	Week 11: Fighting Fatigue with Exercise	Week 21: Fighting Fatigue with Exercise
		Week 22: Too Pooped to Make Healthy Diet Choices?
Week 12: Healthy Eating Check-In	Week 12: Exercise Check-In	Week 23: Exercise Check-In
		Week 24: Healthy Eating Check-In
Week 13: Why Is Enjoyment Such an Important Part of a Healthful Diet?	Week 13: Enjoying Exercise	Week 25: Enjoying Exercise
		Week 26: Why Is Enjoyment Such an Important Part of a Healthful Diet?
Week 14: Need a Break From Stress?	Week 14: Managing Stress with Exercise	Week 27: Managing Stress with Exercise
		Week 28: Need a Break From Stress?
Week 15: The Urge to Eat: Is it Hunger or habit?	Week 15: Celebrate Your Accomplishments	Week 29: Celebrate Your Accomplishments
		Week 30: The Urge to Eat: Is it Hunger or habit?
Week 16: Avoid Pitfalls when Socializing with Others	Week 16: Restarting Exercise After Injury or Illness	Week 31: Restarting Exercise After Injury or Illness
		Week 32: Avoid Pitfalls when Socializing with Others
Week 17: Your Expectations, Thoughts and Beliefs Can Influence Your Success!	Week 17: Expecting the Best From Exercise	Week 33: Expecting the Best From Exercise
		Week 34: Your Expectations, Thoughts and Beliefs Can Influence Your Success!
Week 18: Unhelpful or Negative Thoughts Can be Bad for Your Health	Week 18: Overcoming Unhelpful Thoughts	Week 35: Overcoming Unhelpful Thoughts
		Week 36: Unhelpful or Negative Thoughts Can be Bad for Your Health
Week 19: Getting Back on Track After a Setback	Week 19: Dealing with Setbacks	Week 37: Dealing with Setbacks
		Week 38: Getting Back on Track After a Setback
Week 20: Are Supplements Really Good for You?	Week 20: Choosing Your Exercise Environment	Week 39: Choosing Your Exercise Environment
		Week 40: Are Supplements Really Good for You?
Week 21: Everybody Needs a Good Role Model!	Week 21: Finding Exercise Role Models	Week 41: Finding Exercise Role Models
		Week 42: Everybody Needs a Good Role Model!
Week 22: Important Strategies for Staying on Track	Week 22: Preventing Exercise Setbacks: A Review of Strategies	Week 43: Preventing Exercise Setbacks: A Review of Strategies
		Week 44: Important Strategies for Staying on Track
Week 23: Final Healthy Eating Check-In	Week 23: Exercise Check-In	Week 45: Final Exercise Check-In
		Week 46: Final Healthy Eating Check-In
Week 24: Graduation!	Week 24: Congratulations on Completing the Exercise Program	Week 47: Congratulations on Completing the Exercise Program
		Week 48: You Did It!

### Recruitment, eligibility and consent

AMPLIFI recruits survivors of obesity-related cancers that are associated with a 5-year cancer-free survival of at least 70% (i.e., early stage multiple myeloma and non-Hodgkin lymphoma, localized renal and ovarian cancer, and loco-regional cancers of the colorectum, prostate, endometrium, thyroid, and female breast) [44]. Cancer type and stage is confirmed via cancer registries, health systems or private oncologists. To be eligible, survivors must have completed primary cancer treatment (surgery, radiation, immuno- or chemo-therapy), and show no evidence of progressive cancer or recurrence (exceptions: prostate cancer patients on active surveillance, continuing chemotherapy for non-solid tumors, and recurrences based on blood borne detection methods). Other inclusion criteria are as follows: 1) age > 50 years; 2) Body Mass Index (BMI) between 25 and 50 kg/m^2^; 3) suboptimal levels of physical activity (i.e., < 150 min/week of aerobic activity); 4) reside in an area that receives wireless coverage; 5) English-speaking and writing; and 6) 8th grade educational level (or beyond). Individuals are excluded from participation if enrolled in another diet, weight loss or exercise program, residing in an assisted or skilled nursing facility, or reporting any contraindications to unsupervised physical activity (e.g., balance or mobility issues requiring walkers or wheelchairs, oxygen use, recent myocardial infarction, impending knee or hip surgery, blood pressure > 160/100 if not cleared by treating physician) or participation in telephone- and virtual- assessments and intervention protocols (e.g., severe hearing or vision loss, unable to identify a partner who can provide assistance during virtual assessments [Zoom®, San Jose, CA]) [45], unwillingness to use email, be randomized or complete other study requirements, e.g., assessments).

Recruitment efforts for AMPLIFI began in the fall of 2019 but were thwarted by COVID-19. Under a separate report, we detail both the findings from formative research [18] and protocol modifications made in response to the receptivity and need for virtual assessments [45]. These findings and circumstances not only guided the decision to convert in-person home-visit assessments to virtual means, but also substantially influenced our accrual strategies and indirectly expanded our reach. Hence, we are now able to offer the trial to cancer survivors throughout the continental U.S.

To make-up for lost time incurred by the pandemic, a nationwide, multi-pronged recruitment approach is being implemented based on methods shown previously to be effective in recruiting cancer survivors for lifestyle intervention trials, e.g., state or hospital cancer registry-based identification of cases with subsequent mailing and telephone follow-up [46]. These strategies are being supplemented with solicitations through traditional and social media (brochures, television segments, radio, public service announcements, website [www.amplifymyhealth.org/info], Instagram [https://instagram.com/amplify.survivor.health], and Facebook [www.facebook.com/AmplifySurvivorHealth]), direct emails to University of Alabama at Birmingham (UAB) patients, and outreach via support groups (e.g., Crossroads4hope, Facing Hereditary Cancer Empowered [FORCE], Brenda’s Brown Bosom Buddies) and other organizations (Abroms-Engel Institute for the Visual Arts, Smith Center for Healing and the Arts, O’Neal Comprehensive Cancer Center at UAB, UAB Minority Health & Health Disparities Research Center). To address the needs of underserved cancer survivors, AMPLIFI aims to recruit a sample that is at least 40% racial/ethnic minority, 50% rural, and 60% age 65 and over through oversampling subgroups in registry-directed solicitations and targeted social media. Rural status was defined based on zip codes and the 2010 Urban Area to ZIP Code Tabulation Area Relationship File [47].

Individuals identified as willing and eligible to participate in AMPLIFI are emailed an electronically-generated consent form via Adobe-sign® (San Jose, CA) with an option to complete this process via mail-delivered print copies. Research staff schedule appointments to review the form and ensure that all questions are addressed prior to obtaining signed consent.

### Assessments

Data on clinical and demographic characteristics (e.g., cancer treatment, race/ethnicity, age, and marital, educational and income status) are collected from cancer registries/medical systems or participants at enrollment. Assessments are conducted occur every 6 months for up to 2 years and include five components: 1) a 2-day dietary recall conducted by telephone [48]; 2) collection of accelerometry-based measures (e.g., physical activity and sleep); 3) anthropometric and physical performance testing; 4) collection of biospecimens, and 5) a phone or online survey. Measures are detailed in Table 2.

**Table 2:** Outcomes

**PRIMARY OUTCOMES**	
Diet Quality	Dietary recalls of a non-consecutive weekday and weekend day will be performed via telephone using a multipass method and the Nutrition Data Systems for Research (NDSR) software [48]. The 2-days are averaged at each time point and Diet Quality calculated using the Healthy Eating Index (HEI)-2015, a tool used successfully in a broad range of populations (e.g., minority, older, cancer survivors), [49] will serve as a primary outcome for Project 3 as well as secondary outcomes for Projects 1 & 2, along with other data outcomes, e.g., energy intake and nutrient density.
Objectively-Measured Aerobic Physical Activity	Actigraph accelerometers (Fort Walton, FL) objectively capture physical activity over 7 days and are downloaded and processed using manufacturer procedures and software, and methods similar to those we have reported previously [11]. Moderate-to-vigorous activity assessed using these methods will serve as a primary outcome for Projects 2 and 3, and a secondary outcome for Project 1.
Weight	Measured in light clothing without shoes. The scale dial is captured on Zoom®, first as a “zeroed” value (prior to weighing) and then as the participant weighs. The assessor verifies the weight with both the participant and partner; the process is repeated and the average taken as a primary outcome for Projects 1 and 3 (secondary for Project 2).
**SECONDARY OUTCOMES**	
Other Anthropometric Measures	- Height (self-reported) - Waist circumference: Participant bares midriff to camera and places one end of the ribbon on umbilicus. Partner encircles the waist with the ribbon. The assessor then assures the ribbon is parallel to the floor and flat against the skin as participant rotates in front of camera. Upon exhale, the partner uses a felt-tip marker to mark the ribbon at the point of overlap [50, 51]. The process is repeated with the second ribbon; both ribbons are returned to the study office for measurement and averaging
Physical Performance Testing	The Senior Fitness Battery objectively assesses physical performance in several domains, is sensitive to change, devoid of ceiling effects, and has normative scores [52]. Typically done in-person, tests were adapted to virtual use, refined, and then evaluated for validity and reliability [53]; arm curls and grip strength, were not included given requirements for costly equipment and/or excessive postage. - 30-s chair stand (lower body strength): A standard 18″ unpadded chair is used for this test, though if the participant does not have one, this is recorded and the identical chair is used for follow-up assessments. The participant sits in view of the camera and is instructed to cross arms with hands on shoulders. Upon the assessor’s signal to start, the participant stands up and sits down as many times as possible during a 30-s timed period. - 8′ Get Up & Go test (agility, dynamic balance) Participant starts seated with crossed arms and hands on shoulders while the partner places a sticker and the end of the 8’cord (from mailed supplies) beneath the toe, drawing-out the cord to its full extension in front of the chair. The endpoint is marked by a soccer cone and the cord removed. After positioning the camera to capture the full course and with a focus on the chair (starting and ending points for this test), the participant is given the signal to start. The participant stands up, walks as fast as possible (without running) around the cone, returns to the chair, and sits down. The test is timed using the video – starting from the sign of movement until seated again. - 8′ Walk (gait speed) The chair is removed and the participant starts standing with their toe on the sticker (see test above). Upon the signal to start, they walk as fast as possible through the 8′ point marked by 2 soccer cones (another cone is used to increase visualization) and the camera is focused on the finish-line. This test also is timed using the video – starting from the sign of movement until the finish line is crossed. - Sit-and-reach (flexibility) Seated on the edge of the chair, the participant extends one leg so heel remains on floor, the knee is fully extended and the toe pointed to the ceiling. With the camera capturing the side view of the participant, they are instructed to overlap their hands and extend them towards the toe. The partner measures the distance from the middle finger to the big toe with a vinyl tape measure. Positive for over-reach, negative for under-reaching, zero for touching. - Back scratch (flexibility) While standing with their back toward the camera, the participant reaches arm out and over same shoulder, reaches other arm directly back and attempts to clasp other hand. The partner measures the distance between closest fingers (scoring identical to the test above). - 2-min step test (endurance) The partner is instructed to palpate the participant to locate the iliac crest and then uses the vinyl tape measure to record the distance to the top of the patella which is called-out to the assessor. The assessor calculates the midpoint which is denoted by a sticker. The partner is then asked to measure the distance from the sticker to the floor and call-out the value to the assessor. The assessor records this value for future testing and then instructs the partner to measure this distance against a wall and to mark it with another sticker. Upon the command to start, the participant “marches in place” for 2 min making sure to bring their knees up to point of the sticker. The participant is instructed not to talk, and to take breaks and steady themselves against the wall should they need to while timer continues (the partner also is instructed to “spot” the participant as needed). The number of steps reaching the mark are counted.
Balance Testing	Side-by-side, semi-tandem and tandem stance balance testing as per the Centers for Disease Control protocol is captured on Zoom® [54]. To reduce ceiling effects, the latter test is extended for up to 2 min (or until the stance is broken). This test is also performed near a wall should the participant need to steady themselves.
Blood Pressure	Participant is instructed to sit quietly in front of the camera for 5 min (during which time the assessor turns-off the Zoom® video). Once resumed, the partner is instructed to place the cuff of the automated sphygmomanometer on the participant and to press start (making sure the camera is focused on the display). The assessor reads the values and verifies them with the partner. Blood pressure is standardly assessed in the upper right arm, unless there are contraindications, such as surgery or radiation therapy on the right side; left side assessments are noted and repeated for follow-up assessments.
Muscle Mass	As in the Osteoporotic Fractures in Men (*MrOS*) *Study,* the deuterium creatine (D_3_Cr) dilution method is used that capitalizes on a stable, non-radioactive isotope, to assess muscle mass remotely [55–57]. Participants are provided with a 30 mg capsule of D3Cr and instructed to take the capsule 3 days prior to their assessment date. The night prior to assessment, they begin fasting and continue fasting until they produce the second urinary void of the day. During this void, a test strip is used. The strip is frozen (0 C^o^ or below) until analyzed, using methods originally reported by Clark et al. and adapted by Evans et al. [55–57].
Circulating Biomarkers^a^	Assessor guides the participant in obtaining 5 blood spots on a designated card, which is then dried thoroughly, inserted into a foil pouch with desiccant and frozen (0 C^o^ or below) until analyzed. DBS eluents are batch-tested against known standards for Thyroid Stimulating Hormone (TSH), insulin, glucose, leptin, adiponectin, high density lipoprotein and total cholesterol, triglycerides, interleukin-6, c-reactive protein and tumor necrosis factor alpha at the University of Washington as described previously [58]. Values are expressed in plasma equivalent terms.
Patient Reported Outcomes^a^	*Physical Activity:* As accelerometry provides only activity counts, two common instruments are used to capture data on the various types of physical activities the participants engage in: 1) Godin Leisure-Time Exercise Questionnaire (GLTEQ) is administered given its ease of use and excellent reliability and validity in cancer survivors [59]. An updated version that includes frequency and average minutes of duration of exercise/week within intensity categories (strenuous, moderate [including strength training] and mild) was selected for the current study [60]; and 2) Global Physical Activity Questionnaire (GPAQ) is a 16-item instrument developed for the World Health Organization that assesses physical activity within the contexts of recreation, work and travel. It also measures sedentary behavior and has proven validity and reliability across a broad spectrum of populations [61]. *Cancer-related Outcomes:* The PROMIS Cancer-Related Item Bank is used to assess global physical, mental and social health, perceived stress, and QOL in specific domains, i.e., depression, anxiety, fatigue, sleep, and pain [62]. This tool was synthesized from previous QoL instruments (FACT, EORTC, etc.) and relies on item response theory and complex algorithms to pinpoint the most relevant items in each domain, while reducing the number of items assessed (patient burden). This measure will be completed over the phone or self-administered online, as in past studies [63]. *Quality of Life:* QOL will be measured with the PROMIS global health scale and the EuroQOL-5D-5L (EQ-5D-5L) The EQ-5D-5L assesses 5 dimensions (Mobility, Self-care, Pain/Discomfort, and Anxiety/Depression) [64] at 5 levels of severity: its scores of this instrument are used to calculate Quality Adjusted Life Years. *Health Care Utilization* the validated instrument of Ritter et al. has test-retest reliability of 0.76–0.97 and will capture physician and emergency room visits, and hospitalizations [65], and will be amended to include out-of-pocket costs related to medical visits, and costs of prescribed and over the counter medications [66]. *Comorbidity:* The Older Americans Resources & Services (OARS) Comorbidity Index (43-items) used in multiple studies in older adults will assess the number of chronic medical conditions and symptoms and their functional impact (severity). In addition to serving as an outcome, comorbidity at baseline will be evaluated as a potential moderator. Since falls are a particular issue in this population, an item validated by Chen & Janke that assesses falls in the past year also will be included [67].

^a^Some assessment components (e.g., the Global Physical Activity Questionnaire and biomarkers) were added because of the opportunity to participate in the Accumulating Data to Optimally Predict Obesity Treatment (ADOPT) Consortium [68]

Though almost all AMPLIFI participants complete the anthropometric and physical performance tests over Zoom®, some participants may complete assessments in-person if within driving distance of UAB. Remote assessments are scheduled with each participant; prior to the appointment, they receive the following supplies: 1) Automated sphygmomanometer (Omron 3 Wrist Blood Pressure Monitor, Omron Healthcare Inc., Lake Forrest, IL) to measure blood pressure (an appropriately-sized cuff also is provided based on weight status); 2) Actigraph® accelerometer wGT3X-BT (Pensacola, FL) with an activity/sleep log; 3) One 30 mg capsule of D3 creatine, a urine test strip, and instructions to assess muscle mass; 4) A dried blood spot (DBS) kit (collection card, 2 lancets, band-aid, foil storage pouch, and desiccant) to self-collect a blood sample; 5) Two ribbons (55″ × 1″) and a felt-tip marker (to measure-and-mark waist circumference in duplicate); 6) An 8′ length of cord and two stickers (to mark the distance for the 8′ walk and up-and-go performance tests); 7) two plastic orange soccer cones (to increase visualization of distance-walked on virtual assessments); and 8) a 36″ vinyl tape measure and one sticker (to measure step height for 2-min step test). Also, for participants not owning a scale, a digital scale is included. Prior to the visit, participants and their assessment partners are asked to view instructional videos on performance tests developed by the AMPLIFI team (https://youtu.be/lbxctNuOgLk), collection of DBS (https://youtu.be/lBPLS4PoHv4) and D3 creatinine (https://youtu.be/6BpUgdnPh1c).

Virtual protocols were assessed for reliability and validity, and assessors were trained and evaluated for accuracy prior to initiation [53]. Zoom® sessions are recorded to permit accuracy for timed performance testing, reduce the discrepancy introduced by differential transmission of sight and sound and inform periodic quality assurance evaluations among assessors. Once assessors review these files, time the tests, and log the data, and quality assurance tests are completed, the recordings are deleted. The assessment is repeated in the same modality every 6 months.

**Fig. 2:**
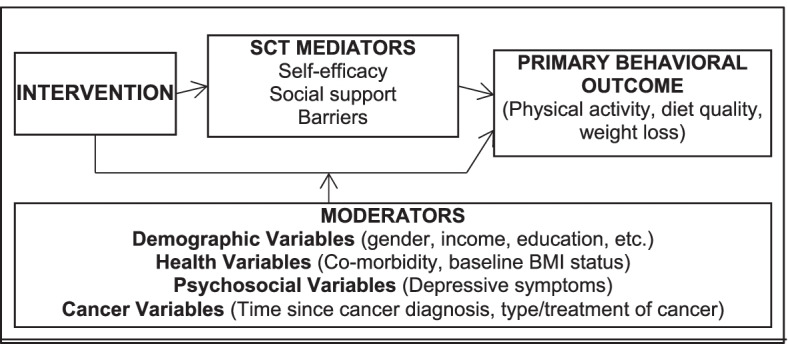
Conceptual model

The assessment survey is designed to collect secondary patient-reported outcomes (Table 2) and the mediators of intervention effects. As Social Cognitive Theory (SCT) provides the behavioral theory framework for the AMPLIFI interventions [19] (Fig. 2), key constructs of SCT are measured at assessment points (baseline, 6, 12, and 18 months). Constructs include: 1) Self-efficacy: The 20-item diet-related instrument (specified in relation to high calorie foods) of Clark et al. [69] and the walking (task) self-efficacy scale of McAuley et al. [70] (abbreviated to 5 items ranging from confidence in walking 10, 20, 30, 40, or 50 min) are used given excellent internal consistency (α = 0.70–0.95) and validated use in cancer survivors. In addition, the 8-item barriers self-efficacy scale for aerobic exercise [71] is used and adapted to include 7-items for resistance exercise; 2) Social support: The validated 5-point scales for exercise and eating a healthy low calorie diet from Sallis et al. [72] which have acceptable test-retest reliabilities (r = 0.55–0.86) and internal consistencies (α = 0.61–0.91) are also employed; and 3) Common barriers to exercise (21 items), resistance training (11 items), and low calorie foods (10 items) are assessed using 5-point Likert-scaled instruments [34, 36, 73, 74].

Questions on health events, falls, and health care utilization are included at each assessment time point as well as midway between assessments (i.e., every 3 months). This information is used to assess potential adverse events. Participants are also asked to report any adverse events immediately to the team using the toll-free study number or study email address. At completion of the intervention, telephone debriefings are performed to capture program satisfaction and solicit suggestions for improvement.

### Randomization

After completion of the baseline assessment, and using a fixed block design, all participants (*n* = 652) are randomized to the sequenced (*n* = 326) or the wait-listed/simultaneous intervention arms (*n* = 326). Sequenced arm participants are then immediately randomized to diet or exercise intervention sequences (*N* = 163 each) for projects 1 and 2. The study flow is depicted in in Fig. 1. The randomization sequence was generated using SAS (Version 9.4, Cary, NC) by an off-site biostatistician. Arm assignment is managed by a central office of blinded staff who have no contact with study participants and implement the computer-generated randomization sequence as baseline assessments are completed.

Participants who are randomized to the wait-list control (followed by the simultaneous intervention), receive brief monthly modules on survivorship topics during the 6-month period they are wait-listed. These modules use the same web-based platform as the other AMPLIFY interventions. Topics are: 1) Good Communication with Physicians; 2) Importance of Quality Sleep; 3) Cognitive Functioning; 4) Benefits of Laughter; 5) Benefits of Creative Arts; and 6) Welcome to the AMPLIFY diet and exercise intervention. This last module serves as an introduction to the simultaneous intervention and addresses next steps and expectations.

### Retention

Retention is addressed at the participant, researcher, and contextual levels [75]. At the participant level, a series of communications was developed to convey appreciation for participation in the trial, e.g., e-cards with brief appreciation videos from investigators at regular intervals, birthday e-cards, and mailed Happy New Year cards. The AMPLIFY logo was incorporated into the design of all communications to ensure easy recognition by participants. At the contextual level, a tracking system shared by assessors and recruiters (who also schedule follow-up assessments) was developed to ensure consistent communication with each participant. At the researcher level, investigators (MP, MM, WDW), recruiters, assessors, and project managers, meet weekly to review recruitment and retention numbers to ensure that participants at risk of drop-out are identified early. Changes to procedures and communications with participants are discussed at these meetings to ease participant burden and reduce drop-out risk. Participants who may need to pause the intervention due to health or personal reasons (e.g., COVID-19, elected/emergency surgeries, deaths in the family, changes of residence, prolonged vacations) are also discussed by project managers and investigators (WDW, LR) to determine when they can resume participation. Outcome data will be collected from all participants, including those who discontinue or deviate from the intervention protocols and are still willing to participate in assessments.

### Statistical considerations

#### Data management, safety and monitoring

Data will be entered into REDCap, a password -protected secure web application for building and managing online surveys and databases with built in range checks for data values and other processes to promote data quality. To protect confidentiality of data, information will be collected from the Web-based surveys, personal identifiers will not be directly linked with the data collected, nor used in any reports, materials, or presentations that emanate from this work. All electronic files containing personal identifiers will be stored only on study staff computers at UAB’s on-site file servers (located behind secure firewalls). Information transferred to the server (for backup purposes) will be done via secure file transfer protocol. Files may be transferred to other computers via the Internet. When this is done, the files will be protected through a method that encrypts their contents during transfer and storage. The on-site file servers are physically accessible only to network support specialists (locked rooms). The on-site file server will be electronically accessible only to study staff and authorized contractors (as part of the research team—not necessarily UAB Employees) through user/password protection. Non-electronic files (i.e., consent forms) will be kept in a locked file cabinet, located in a locked room. Any biological samples will only be labeled with study identification (ID) numbers and the time and date of collection. Access to this room is limited to research staff only. In addition, all research staff are HIPAA certified and have completed and are current with regard to IRB training. Data sets provided as a function of the NIH Data Sharing Requirement will be stripped of identifying information according to HIPAA policy.

An external data safety and monitoring board (comprised of oncology and clinical trial investigators, clinicians, patient advocates; independent from the sponsor and competing interests) and UAB Scientific Monitoring Subcommittee will meet annually to audit trial conduct and review findings, scientific findings, IRB compliance, any adverse events, etc.

#### Sample size and power

Power calculations are based on testing the primary aims for each project, resulting in sample sizes of 130 participants for each of the intervention arms in Projects 1 and 2, and 260 participants for the wait-list control/simultaneous arm. Power calculations for Projects 1 and 2 assume a two-sided two-group chi-square test for groups with unequal sample sizes, and those for Project 3 assume a two-sided two-group chi-square test for groups with equal sample sizes and a 5% significance level. For Project 1, assuming 130 participants for the intervention arm and 260 participants for the wait-list control arm, and that 43.9% of the diet intervention arm and 27.3% of the control arm will lose ≥3% weight yields 90% power to detect a difference of ≥16.6% between study arms as statistically significant [28]. For Project 2, assuming 130 participants for the intervention arm and 260 participants for the wait-list control arm, and increases of participants meeting recommendations of 17.6% for the exercise intervention arm and 3.8% for the control arm based on the BEAT Cancer efficacy trial yields > 95% power to detect a difference of ≥13.8% between study arms as statistically significant [12]. For Project 3, assuming 260 participants/arm and increases in the proportion achieving primary behavioral goals (i.e., improved diet quality, ≥3% weight loss, and ≥ 150 weekly minutes of ≥ moderate intensity aerobic physical activity) of 33.3% for the sequenced arm and 22.2% for the simultaneous arm based on past sequential and simultaneous interactive computer-tailored interventions [76] yields 80% power to detect a difference of ≥11.1% between study arms as statistically significant. Accrual targets assume 20% drop-out. If study dropout for Project 3 is greater than anticipated, we will still have adequate power to detect modest differences between the two study arms. For example, if this project concludes with 200 participants/arm, there will be 80% power to detect a difference of ≥12.5% between study arms as being statistically significant; if the project concludes with 136 participants per arm, there will be 80% power to detect a difference of ≥15% between study arms as being statistically significant.

#### Data analysis

Descriptive statistics will be calculated for all study variables, and normality checks will be performed with subsequent data transformation if indicated prior to data analysis. Statistical tests will be two-sided using a significance level of 5%. Statistical analyses will be conducted using SAS version 9.4 or higher (Cary, NC). Multiple imputation methods may be used to address missing data for variables with moderate amounts of missing data. Formal interim analyses and stopping rules are not planned given the relatively short duration of our intervention and primary aims of behavior change.

#### Primary outcomes

An intent-to-treat analysis will be performed for all primary outcomes (Table 2). The chi-square test will be used to test for differences in the outcomes (proportions) between arms. An exact 95% confidence interval based on the binomial distribution will be determined for the binary outcome (proportion). Logistic regression models will then be used to regress the binary outcome on intervention arm, with pre-specified covariates such as age and gender in order to test for arm differences in the covariate-adjusted outcomes (proportions). For Projects 1 and 2, outcomes will be compared at 6 months. For Project 3, outcomes will be compared immediately post-intervention (12 months for the sequenced arm and 18 months for the simultaneous arm).

#### Secondary outcomes

Analyses of secondary outcomes (Table 2) will account for comparisons of the two study arms (between-group comparisons) as well as the baseline and post-intervention measurements of each participant (within-arm comparisons). Since most of these outcomes are continuous, the primary method of analysis for these outcomes will be mixed models repeated measures analysis. This method will allow us to compare changes over time (within-group changes) and differences between groups simultaneously. An appropriate structure for the covariance matrix will be selected based on the final data. The Tukey-Kramer multiple comparisons test will be used as the post hoc test. These models will include terms for group, time, and group x time, as well as terms for any other covariates and interactions that are of scientific interest such as age, sex, race/ethnicity and rural status. Additional general linear mixed models may also be used. Overall unadjusted between-group comparisons at baseline may be performed using the two-group t-test, and overall unadjusted within-group comparisons between two time points may be performed using the paired t-test.

#### Mediation and moderation

A multi-level approach to mediation and moderation analyses will be used to test the hypotheses that greater improvements in self-efficacy, barriers, and social support will be found in the sequential arm (versus simultaneous arm) leading to greater improvement in behavioral outcomes. The multi-level approach examines how the SCT constructs (Fig. 2), measured over time, may influence the effect of the intervention on study outcomes and allow for the estimation of the direct association between the intervention and the outcome variable at both the between and within person levels (% study goals) [77, 78]. Indirect effects (association of intervention through SCT constructs) are estimated as the product of the coefficients linking the intervention to an SCT mediator and the intervention to the outcome [79]. A moderated mediation model will then be examined: this hypothesizes that the indirect effect of the intervention on the outcome is conditional on the level of the hypothesized moderator variable (Fig. 2) [80]. Finally, Monte Carlo bootstrapping will be used to construct 95% confidence and test for significance [81]. MPlus Version 8 software will be used (Muthen & Muthen, Los Angeles, CA) [82]. Mediation and moderation analysis will similarly be pursued using data collected at baseline and 6-month follow-up for Projects 1 and 2, though exploring effects that solely focus on either weight loss or increases in physical activity, respectively.

#### Economic analysis

Intervention implementation costs and participants’ health care costs (based on self-reported health care utilization) will be calculated. Intervention implementation costs include mostly those of personnel for website management and updates, and supplies. Development and research-related expenses will not be included. We will also estimate intervention costs for participants, i.e., time spent on the intervention web sessions. Health care costs will be compared across arms to determine if any of the AMPLIFY interventions result in health care cost savings. We will conduct a within trial cost-effectiveness analysis to compare intervention implementation costs net of health care cost savings to the interventions’ effectiveness measured by the gain in quality-adjusted life years (QALYs) [83, 84]. QALYs will be estimated based on the utility scores of the EQ-5D [85–87]. As for the main efficacy assessment, we will compare the dietary intervention versus wait-list control (Project 1), the exercise intervention vs wait-list control (Project 2), and the sequenced vs combined interventions (Project 3). Incremental Cost-Effectiveness Ratios (ICERs) will be calculated and compared to commonly used thresholds (e.g., $50,000 per QALY) to determine which interventions are worth their costs [88]. Sensitivity analyses will examine robustness of results to assumptions such as unit cost values used for salaries or health care events, and separate analyses will examine cost-effectiveness for younger/older, rural/urban, minority/non-minority survivors.

### Dissemination plan

Investigators will communicate trial results to participants, healthcare professionals, the public, and other relevant groups via publications (in peer-reviewed scientific journals, study newsletters), presentations (to community organizations, professional societies), and reporting in Clinicaltrials.gov.

## Discussion

The current study will test the adaptation of efficacious interventions to web-based platforms for cancer survivors. It is important to determine the extent to which programs originally conceived in a research setting are amenable for wide dissemination and maintain their potential to improve survivors’ outcomes via remote delivery. This study also will be the very first to test the relative efficacy of simultaneous versus sequential approaches to promote multiple behavior change among cancer survivors. Past studies assessing multiple behavior changes in different populations have produced mixed findings [14], with some results supporting sequential approaches to multiple behavior change [17, 89], and others favoring simultaneous [90] or showing no differences [91]. Thus, programmatic research to pinpoint optimal sequencing and/or combination of multiple health behavior interventions for specific target populations will help inform future clinical practice and health promotion efforts.

Strengths in the design of this program project include integration amongst the RCTs, which allows for sharing of a control condition and research cores. Other strengths include: objective measurement of weight and aerobic physical activity (as well as the collection of physical performance, muscle mass and key circulating biomarkers); an evidence-based theoretical framework; and formal cost analyses, which will shed light on the dissemination and implementation potential of the AMPLIFI programs. In addition, the expansion of accrual of cancer survivors across the continental U.S. will further bolster the generalizability of results. At its very core, AMPLIFI builds on the success of two previously proven efficacious interventions, by using dissemination and implementation science tools to glean key user and stakeholder input and arrive at scalable interventions. The current study also moves the cancer survivorship field forward by recruiting a large, diagnosis-diverse sample and focusing on older, rural, and racial minority groups, all of which have been underrepresented in the lifestyle intervention research to date [15].

As for study limitations and/or challenges, the COVID-19 pandemic caused an initial delay in participant recruitment and necessitated the adoption of remote assessment protocols [45]. To overcome the challenge of assessment retention over the extended period of study enrollment (i.e., 2 years), we are implementing a comprehensive set of retention activities. To assuage concerns regarding potential differential drop-out between the active intervention and wait-list arms, the wait-list participants receive access to survivorship online videos designed to support health and wellbeing. Similar concerns exist regarding participant preferences or motivational readiness for specific health behaviors. For example, cancer survivors who are assigned the diet intervention first may demonstrate better retention/engagement than those assigned to exercise first. Further, engagement with the website may attenuate over time, due to the duration of the interventions. Thus, participant website engagement will be closely monitored throughout the project with patterns of engagement over time scrutinized to identify vulnerable time points that signal engagement drop off that could be addressed with minimal touch contact in future intervention implementation (e.g., additional text messaging, etc.).

AMPLIFI’s greatest scientific contribution is likely the development and adaption of efficacy-tested diet and exercise intervention content for delivery via interactive web-based platforms (as well as the refinement of remote assessments that are able to evaluate the impact of such programs). The use of innovative technology to enhance the reach of lifestyle interventions has substantial public health implications given the rising numbers of cancer survivors at risk for physical inactivity, poor diet quality and excess weight [92]. Scalable internet-based intervention strategies can help address these quality-of-life concerns, as well as cancer disparities among older, racial/ethnic minority, and rural survivors.

## Data Availability

Data sharing is not applicable to this article as no datasets were generated or analyzed yet for the current study.

## Abbreviations

AMPLIFI: Adapting MultiPLe behavior Interventions that eFfectively Improve (AMPLIFI) cancer survivor health
RENEW: Reach-out to ENhancE Wellness
BEAT: Better Exercise Adherence after Treatment for cancer
U.S.: United States
RCTs: Randomized controlled trials
QALYs: Quality adjusted life years
IRB: Institutional Review Board
SCT: Social Cognitive Theory
AMPLIFY: AiM, Plan, and act on LIFestYles
Kg: Kilogram
M: Meters
S.M.A.R.T.: Specific, Measurable, Achievable, Relevant, and Time-Bound
COVID-19: Coronavirus Disease 2019
UAB: University of Alabama at Birmingham
FORCE: Facing Hereditary Cancer Empowered
Mg: Milligram
DBS: Dried blood spot
ID: Identification
HIPAA: Health Insurance Portability and Accountability Act of 1996
NIH: National Institutes of Health
ICERs: Incremental Cost-Effectiveness Ratios
